# Hydrogen Gas Sensors Based on Semiconductor Oxide Nanostructures

**DOI:** 10.3390/s120505517

**Published:** 2012-04-30

**Authors:** Haoshuang Gu, Zhao Wang, Yongming Hu

**Affiliations:** Faculty of Physics and Electronic Technology, Hubei University, Wuhan 430062, China; E-Mails: wangzhaohubu@163.com (Z.W.); yongming.hu09@gmail.com (Y.H.)

**Keywords:** hydrogen gas sensor, semiconductor oxides, nanostructure, thin films, one-dimensional nanostructures

## Abstract

Recently, the hydrogen gas sensing properties of semiconductor oxide (SMO) nanostructures have been widely investigated. In this article, we provide a comprehensive review of the research progress in the last five years concerning hydrogen gas sensors based on SMO thin film and one-dimensional (1D) nanostructures. The hydrogen sensing mechanism of SMO nanostructures and some critical issues are discussed. Doping, noble metal-decoration, heterojunctions and size reduction have been investigated and proved to be effective methods for improving the sensing performance of SMO thin films and 1D nanostructures. The effect on the hydrogen response of SMO thin films and 1D nanostructures of grain boundary and crystal orientation, as well as the sensor architecture, including electrode size and nanojunctions have also been studied. Finally, we also discuss some challenges for the future applications of SMO nanostructured hydrogen sensors.

## 1. Introduction

Hydrogen is regarded as one of the best clean energy carriers, which is the ultimate fossil fuel candidate, with a high heat of combustion (142 kJ/g), low minimum ignition energy (0.017 mJ) and wide flammable range (4–75%), as well as high burning velocity. The combustion product of hydrogen is water, which is free from contamination and can be converted into hydrogen and oxygen again for periodic duty. Liquid hydrogen has already been used for rocket fuels. Moreover, hydrogen is also used in metal smelting, glassmaking, semiconductor processing, petroleum extraction and the daily chemical industry, *etc.* due to its strong reducing properties. In addition, hydrogen can also be applied in biomedical, environmental protection and seismic surveillance area such as for indicating certain type of bacterial infection, detection of environmental pollution, *etc.*

Hydrogen gas is tasteless, colorless and odorless so it cannot be detected by human beings. The low ignition energy and wide flammable range makes it easy inflammable and explosive. Therefore rapid and accurate hydrogen detection is necessary during the production, storage and use of hydrogen and it is also essential for monitoring/controlling the hydrogen concentration of nuclear reactors, coal mines and semiconductor manufacturing, *etc.* [1–3].

As the traditional hydrogen detectors such as gas chromatographs, mass spectrometers and specific ionization gas pressure sensors are limited by their large size, expensive cost and slow response and sometimes high temperature of use, with potential safety hazards, hydrogen gas sensors of smaller size, lower production cost and power consumption, as well as lower operation temperature and faster response are required for widespread use such as portable and *in-situ* monitoring, and the rapid development of the hydrogen economy has promoted research on new types of hydrogen gas sensors with more rapid and accurate hydrogen sensing, near room temperature (RT) operation without power sources and compatibility with microelectronic integrated circuits.

**Figure 1.**
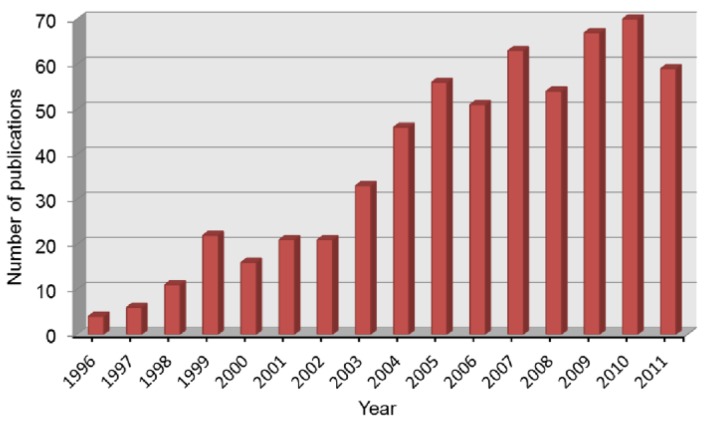
Publications about semiconductor hydrogen sensors since 1996 according to an enquiry in Thomson Reuters ISI Web of Knowledge.

As present, there are many types of the commercially available hydrogen sensors, including electrochemical, semiconductor, thermoelectric, metallic, optical and acoustic ones, *etc.* Among them, semiconductor sensors exhibit high sensitivity, fast response, long-term stability and potential for integration in hydrogen sensing performance [4–7]. Figure 1 shows the growing number of relevant publications about semiconductor hydrogen sensors. This type of hydrogen sensors still suffers however from high operation temperatures, which results in high power consumption and potential safety hazards. Moreover, the cross selectivity to other combustible or reducing gases is another critical issue, which should be restricted to enhance the sensing accuracy.

In recent years, semiconductor nanostructures such as nanowires (NWs) and thin films have been employed as sensing materials for building high-performance hydrogen sensors due to their high specific surface area and novel electron transportation properties. For instance, mesoporous thin films or nanotube arrays synthesized by anodic oxidization shows enhanced hydrogen sensing properties compared to traditional film-based sensors due to the greater specific surface area [8–14]. As for the dimensional reduction, 1D nanostructures show much higher sensing performance such as higher sensitivity, broader limit of detection (LOD), lower operation temperature and response time than thin films [15,16]. In addition, nanoparticles-decoration semiconductor nanostructures have also been widely investigated for increasing of sensitivity and selectivity to hydrogen gas [17,18].

In this review, the recent research efforts made in gas sensors based on semiconductor oxide (SMO) nanostructures are comprehensively reviewed. Among them, the research works on thin film and 1D nanostructures reviewed in this article are mainly published in the past five years. In Section 2, the sensing mechanism of hydrogen sensors based on metal oxide semiconductor nanostructures and their critical issues will be introduced. In Section 3, the recent research progress of hydrogen sensors based on SMO thin film and 1D nanostructures will be reviewed, respectively.

## 2. Classification and Critical Issues of SMO Hydrogen Sensors

### 2.1. Types of SMO Hydrogen Sensors

As a hydrogen detecting device, the hydrogen sensor is essentially a transducer that transforms the variation of physical or chemical properties into an electrical signal for practical applications. According to the variation of electrical and optical properties of SMOs under a hydrogen-containing atmosphere, the SMO hydrogen sensors can be divided into four types: resistance based, work function based, optical and acoustic sensors.

#### 2.1.1. Resistance-Based

In 1950, Wagner *et al.* reported the variation of electrical properties when ZnO is exposed to reducing gases [19]. After that, a series of research works about the sensing behavior of SMO to reducing gases were reported by Seiyama, *et al.* since the 1960s [20]. Up to present, many kinds of SMO were investigated as hydrogen sensing materials, including SnO_2_, ZnO, TiO_2_, Nb_2_O_5_, In_2_O_3_, FeO, Fe_2_O_3_, NiO, Ga_2_O_3_, Sb_2_O_5_, MoO_3_, V_2_O_5_ and WO_3,_ which exhibit large variations in resistance after exposure to hydrogen gas. Among them, SnO_2_ and ZnO are the mostly used SMOs in the resistance type hydrogen sensors. For instance, several studies have been carried out on the hydrogen sensing properties of SnO_2_ and ZnO 1D nanostructures as resistance type sensors since the first report on the gas sensing performance of SnO_2_ nanobelts [21,22].

**Figure 2.**
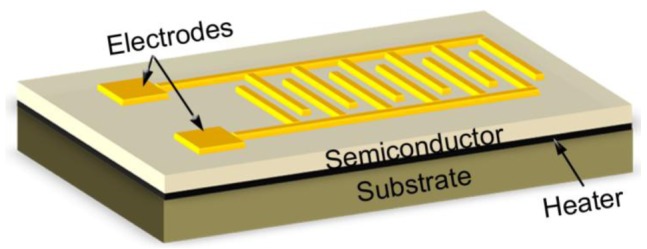
Schematic of a resistance based SMO hydrogen sensor.

Figure 2 shows the typical structure of a resistance based SMO hydrogen sensor, which consists of a SMO layer on an insulating substrate and two electrodes, as well as a heater under the sensitive layer. During operation the sensitive layer will be heated to a certain temperature for enhancement of the sensing performance. This temperature, which depends on the sensitive oxide materials used, is typically several hundred degrees Celsius. The resistance (*R*) of the sensitive layer will change due to the exposure to hydrogen gas. The variation depends on the hydrogen concentration and exhibits an approximately linear relationship with the hydrogen concentration within a certain range.

**Figure 3.**
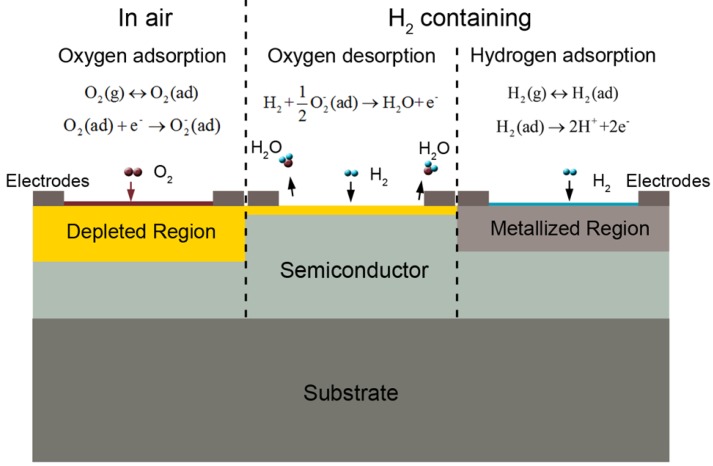
The hydrogen sensing mechanism of resistance based SMO sensors.

The resistance-based sensing mechanism of SMO is complicated, and has been investigated by many researchers. The commonly accepted mechanism is based on the variation of the surface electron depletion region due to the reaction between hydrogen and the chemisorbed oxygen on the surface. As described in Figure 3, under an air atmosphere the oxygen molecules can get adsorbed on the surface of the semiconductor and extracts electrons from the conduction band to form oxygen ions. That may lead to the formation of an electron depletion region near the surface, which can greatly increase the resistance due to the decrease of net carrier density. When the sensor is exposed to a hydrogen atmosphere, the hydrogen molecules will react with the adsorbed oxygen species. The redox reaction is exothermic and results in the fast desorption of produced H_2_O molecules from the surface. The released electrons will reduce the thickness of the depletion region, and decrease the resistance of the semiconductors. When the sensor is exposed to the air ambient again, the depletion region will be rebuilt by adsorbed oxygen species. The resistance will regain the initial level before hydrogen response.

Moreover, the surface chemisorption of dissociated hydrogen may also play an important role in the hydrogen sensing behavior. As reported, hydrogen dissociated on the surface of a semiconductor induces an intermediate energy level for the transfer of charges from hydrogen to the conduction band. An accumulation layer of electrons is therefore created and results in a metalized region near the surface, which may greatly decrease the semiconductor resistance. When the hydrogen ambient was removed by air, the accumulation layer of electrons would be eliminated, and this leads to the recovery of resistance. Wang *et al.* have studied the hydrogen-induced metallization of ZnO and found that the ZnO surfaces and NWs became metallic when surface oxygen (O) atoms were saturated by hydrogen [23]. Xu *et al.* have also demonstrated a first-principles study of hydrogen adsorption on ZnO NWs and found that the adsorption with half a monolayer of hydrogen on an oxygen surface induces metallic behavior [24]. This process has also been reported for TiO_2_ based hydrogen sensors [25,26].

#### 2.1.2. Work Function-Based

This type of hydrogen sensors are operated based on the variation of work function induced by hydrogen. The work function based sensors is generally formed by the metal/oxide/semiconductor (MOS) layers. According to the difference in structure, the work function based hydrogen sensors can be divided into three major types: the Schottky diode type, MOS capacitor type and the MOS field effect transistor (MOSFET) type.

##### (1) Schottky Diode

**Figure 4.**
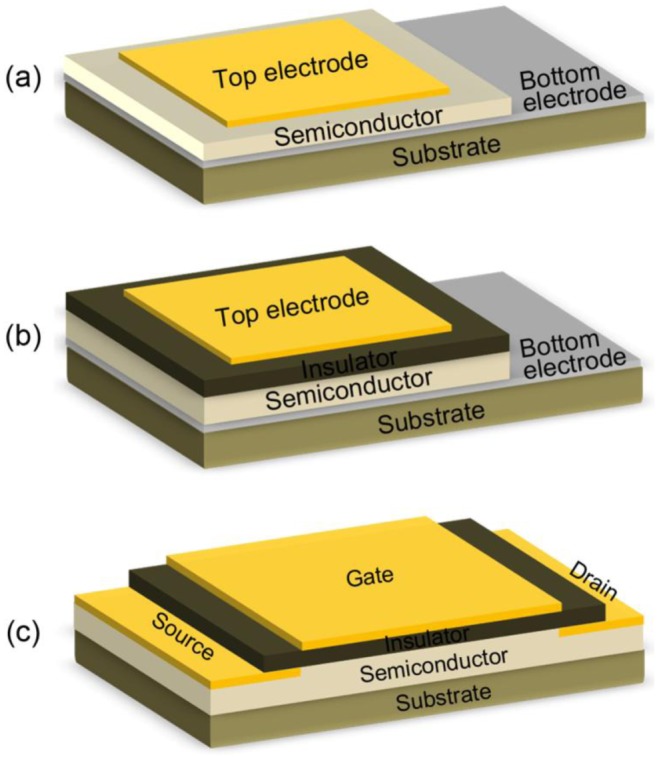
The schematic diagram of work function based SMO hydrogen sensors. (**a**) Schottky diode type; (**b**) MOS capacitor type; (**c**) MOSFET type.

As shown in Figure 4(a), a Schottky diode type hydrogen sensor consists of a metal-(oxide)-semiconductor-metal structure. The top electrode is the gas sensitive metal layer, which forms a Schottky contact at the interface between the metal and semiconductor (or a thin oxide layer deposited on the semiconductor), while the bottom electrode is a metal layer that form Ohmic contact with the semiconductor film, or sometimes a gas sensitive metal layer as well to form the back-to-back Schottky junction structure (mostly used for semiconductor thin film or NW based hydrogen sensors).

Hydrogen molecules can get adsorbed and dissociated into hydrogen atoms in the gas sensitive metal layer (generally Pd, Pt, Au, Ag and Cu, *etc.*). The diffusion of hydrogen atoms may lead to the formation of dipole layer at the interface between metal and oxide, which changes the work function of the metal and results in the variation of Schottky barrier height at the interface. Consequently, the measured voltage in *I-V* curve will be shifted corresponding to the adsorbed hydrogen atoms, which can be used for detecting concentrations. An alternative explanation of the sensing mechanism was carried out by Yamamoto *et al.* by employing a Pd/TiO_2_ Schottky diode sensor [27]. They suggested that the decrease of work function of Pd should be attributed to the reaction of adsorbed oxygen anions on Pd with the hydrogen species. Moreover, Zemel and co-workers attributed that to the hydrogen induced interface states that resulted in the change in electrical properties [28].

Several SMOs were employed to form the Schottky diode sensors, including SnO_2_, TiO_2_, ZnO and Nb_2_O_5_, *etc.* For instance, Lu *et al.* have reported a Schottky diode type hydrogen sensor based on nanostructured SnO_2_ films with Pd electrodes [29]. The sensor exhibited high sensitivity and fast response (less than 10 s) to 100 ppm of hydrogen gas at 300 °C under a forward bias voltage of 0.5 V. Hyodo, Shimizu, Iwanaga and Miyazaki, *et al.* have demonstrated a series of research works about the Pd/TiO_2_ Schottky diodes hydrogen sensor based on nanoporous TiO_2_ films [8,9,13,30]. They suggested that higher sensitivity could be obtained under reverse bias conditions than with forward biased conditions. A high performance Schottky diode type hydrogen sensor was reported by Das *et al.*, which is fabricated based on individual ZnO NW and Pt electrodes and exhibited sensitive and fast response at RT [31]. Similar research works have also been reported by Kim and Shafiei *et al.* [32,33]. Moreover, Hyodo *et al.* also reported a diode type Pd/Nb_2_O_5_ hydrogen sensor based on nanoporous Nb_2_O_5_ films [14].

SMOs were also used to fabricate SiC-based Schottky diode hydrogen sensors. For example, Yu, Kandasamyu, Shafiei, Wlodarski and co-workers have reported several hydrogen sensors based on this structure, including Pt/ZnO-nanorod (NR)/SiC, Pt/TiO_2_/SiC and Pt/SnO_2_-NWs/SiC Schottky diodes [34–37]. These sensors exhibited wide range of operation temperature and obvious work function shifts under forward and reverse biased conditions.

In addition, ferroelectric oxides were also employed as the oxide layer for fabricating hydrogen sensors with catalytic-metal/ferroelectric/metal structures. Although this type of ferroelectric device is always used as a capacitor and was categorized as a capacitor type hydrogen sensor by Hübert *et al.*, their catalytic-metal/ferroelectric/metal structure resemble more closely the diode type [1]. Zhu, Tan and co-workers have demonstrated several ferroelectric thin film capacitor hydrogen sensors since 1998, including (Ba,Sr)TiO_3_ and Pb(Zr,Ti)O_3_ ones [38–40]. All these ferroelectric-based hydrogen sensors show shifted *I*–*V* curves under a hydrogen-containing atmosphere. They attributed it to the lowering of Schottky barrier height through charges induced by hydrogen ions at the interface. Moreover, they suggested that the high permittivity of the amorphous ferroelectric thin films can enhance the proton polarization at the metal/ferroelectric interface and greatly improves the built-up interfacial potential induced by the hydrogen. A detailed review was also carried out by them in 2003 [41]. Besides, Nakagomi also reported a hydrogen sensor based on a K_1−x_Na_x_NbO_3_ thin film in 2005 [42].

##### (2) MOS Capacitor

As shown in Figure 4(b), the MOS capacitor hydrogen sensors are similar in structure to the above mentioned Schottky diode type sensors. However, a thick insulator layer is deposited between the catalytic sensitive metal layer and the semiconductor layer to prevent the current conduction and build up a charge-accumulation layer on both sides. When the sensor is exposed to a hydrogen-containing atmosphere, the dissociated hydrogen atoms can diffuse into the metal/insulator interface and lead to a lateral shift in the *C-V* and *G-C* plots corresponding to the hydrogen concentration.

Steele *et al.* firstly reported the MOS capacitor type of hydrogen sensors in 1976 [43]. After that, a series of research works have discussed silicon-based capacitor hydrogen sensors. In recent years, most of the reported MOS capacitor hydrogen sensors were based on a SiO_2_ insulator with a layer of palladium on top. However, several investigations on MOS capacitor hydrogen sensors based on SMOs were also carried out. For instance, Kang *et al.* have developed a class of MOS capacitor gas sensors in 1994, which employed SnO_x_ and ZnO as the gas-adsorptive oxide layer on silicon [44]. The sensor shows enhanced hydrogen sensing properties at low temperature. They attributed it to the hydrogen dipoles in addition to the reduction of chemisorbed oxygen at the interface. Dwivedi and co-workers have investigated the effect of hydrogen-induced interface traps on a titanium dioxide-based palladium gate MOS capacitor [45]. The device is sensitive to hydrogen (1–3%) at RT, where the interface state density (*D*_it_) increases with the hydrogen gas concentration.

Recently, Yadav and co-workers have demonstrated a Pd/TiO_2_/Si MOS hydrogen sensor, which exhibited larger lateral shift in *C-V* and *G-V* response and short response time (<30 s) with exposure to 4% of hydrogen gas in nitrogen at RT [46]. Weng and co-workers have reported a Pd/TiO_2_/SiO_2_/SiC capacitor gas sensor working at high temperature [47]. They explained the leakage current detecting mechanism by a trap-assisted conduction model that the barrier height can be changed corresponding to the hydrogen concentration.

##### (3) MOSFET

Figure 4(c) shows the schematic diagram of a MOSFET or metal-insulator-semiconductor field effect transistor (MISFET) hydrogen sensor, which is also based on the variation of work function of the catalytic hydrogen sensitive metal (gate electrode) under exposure to hydrogen gas. Typically, the MOSFET device consists of a metal-SiO_2_-Si structure, in which two Si regions were ion-implanted to form the source and drain. Moreover, the Schottky barrier between metal and semiconductor can also be used for building the source and drain junction, especially when the MOSFET is formed based on the 1D nanostructures. After the source is grounded, the conductivity between source and drain can be modified by adjusting the gate voltage (*V*_GS_). When this sensor is exposed to a hydrogen-containing atmosphere, the hydrogen atoms diffused to the interface between metal and insulator can form a dipole layer and thus change the gate voltage, which finally results in the variation of measured voltage signal corresponding to the hydrogen concentration.

Lundström and Stilbert, *et al.* first reported the MOSFET type hydrogen sensor in 1975 [48,49]. Recently, several research works about MOSFET gas sensors based on SMOs were published, while only a few research works about hydrogen sensors have been reported. For instance, Zeng *et al.* demonstrated a H_2_S sensor based on a single In_2_O_3_ NW transistor [50]. Zhang *et al.* reported an In_2_O_3_ NW FET gas sensor with high performance towards NH_3_ [51]. Anderi and co-workers reported a FET device based on an individual SnO_2_ nanobelt, which was modeled as two back-to-back Schottky diodes with a series resistance from the SnO_2_ nanobelt [52]. They suggested that the sensor worked as a FET only when it was exposed to hydrogen-containing condition, where the rectifying behavior of the source and drain contacts disappear, and the resistance of the device could be modulated by the gate voltage.

#### 2.1.3. Optical SMO Sensor

Optical SMO hydrogen sensors are based on the variation of optical properties of SMO materials or the whole sensor when they are exposed to a hydrogen-containing environment. Most optical hydrogen sensors are based on thin films coated onto the tip or side wall of an optical fiber. These optical fiber based hydrogen sensors are always known as optrodes or optodes [53].

Typically, SMO optical hydrogen sensors operate based on the measurement of the evanescent field interaction. The evanescent field is always formed at the boundary between certain different media such as the core of an optical fiber. Evanescent field decays exponentially with the distance from the optical fiber. SMO thin films can be deposited onto a polished optical fiber as a sensing layer. The refractive index of the SMO layer will be changed by the exposure to hydrogen, and result in an attenuation change in the evanescent field, which can be detected as a change in transmittance. Sekimoto and co-workers reported a WO_3_ based evanescent wave hydrogen sensor with Pd and Pt catalyst in 2000 [54]. The refractive index of WO_3_ changed during its reaction with hydrogen, which is coupled to the evanescent field, and results in the variation of the power intensity of fiber with the hydrogen concentration.

#### 2.1.4. Acoustic Sensors

Acoustic hydrogen sensors operate basing on the variation of acoustic wave properties (e.g., resonance frequency) of the piezoelectric materials due to adsorption of hydrogen onto the sensing layers. As known, the resonance frequency of bulk and surface acoustic wave (BAW, SAW) devices is sensitive to the accumulation of mass on the surface of the piezoelectric materials, which is always used to measure the mass of concentration of loading matters in ambient or in liquid conditions and possess ultra-high sensitivity.

**Figure 5.**
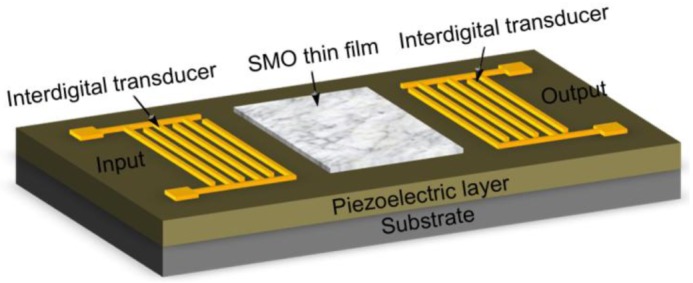
The schematic diagram of a SAW hydrogen sensor.

Figure 5 shows a typical structure of a SAW hydrogen sensor. Two interdigitated transducers (IDTs) were deposited on a piezoelectric film. The input IDT converts the applied electrical signal into acoustic waves by the inverse piezoelectric effect. The generated acoustic waves propagate along the surface within 1 or 2 acoustic wavelengths, and are converted back to electrical signals by the output IDT. The acoustic wave is very sensitive to the variation of surface conditions, and therefore can be used in hydrogen sensing when a SMO thin film hydrogen sensing layer is deposited on the surface of the piezoelectric layer between the IDTs. The hydrogen concentration can be detected by the frequency shift tested on the output port of the SAW device. The piezoelectric materials mostly employed for fabricating acoustic wave devices were LiNbO_3_, LiTaO_3_ and AlN, *etc.*, while WO_3_, ZnO and InO_x_ were often used as SMO sensing layer [55–58].

### 2.2. Critical Issues

Among the reported investigations about SMO-based hydrogen sensors, the sensitivity, response, recovery time, gas selectivity, LOD and the temperature stability were always of concern, while the humidity and long-term stability were seldom investigated.

The sensitivity of resistance type hydrogen sensors can be defined as *S* = *R*_0_/*R*_g_ for *n*-type SMOs and *S* = *R*_g_/*R*_0_ for *p*-type SMOs, while some are defined as the relative change of the resistance *S* = Δ*R/R*_0_ or *S* = Δ*R/R*_g_, where *R*_0_ and *R*_g_ are the resistance measured before and after exposure to hydrogen, respectively. The response and recovery time is always defined as the time needed for the device to undergo a 90% change in the sensitivity in equilibrium upon exposure to hydrogen gas. According to the definition of LOD, it should be the lowest concentration that can be detected with three-times higher than the background signal. Therefore, LOD should be estimated by extrapolating the concentration dependent *S* to 3*σ/R*_0_, where *σ* is the standard deviation of *R*_0_. However, most investigations reported their LOD by directly pointing out the measured concentrations in their experiments, while some do not mention the LOD of their hydrogen sensors. Therefore, we only listed the experimentally measured range of hydrogen concentration in the literatures. Moreover, hydrogen sensors must pick out and measure the hydrogen gas from a complicated ambient in the environment to meet the demands for different applications. However, most of SMOs are sensitive to other reducing gas besides H_2_, such as CO, H_2_S, C_2_HOH and CH_4_, *etc.* Therefore, the improvement of hydrogen gas selectivity is crucial in the R&D of SMO hydrogen sensors. Size reduction, surface modification by nanoparticles and doping are efficient methods for increasing the hydrogen gas selectivity of SMOs.

Temperature is an important factor that greatly influences the hydrogen response of SMO sensors. Usually, higher temperature would lead to higher sensing performance due to the lowering of activation energy for gas adsorption and desorption. However, the sensitivity reached a best value and then decreased with the increasing temperature in some reports [59,60]. This phenomenon was explained as the disappearance of the depletion layers at the grain boundaries under high temperature. In addition, a major object in the research of SMO hydrogen sensors is to decrease the operation temperature (even to RT) with considering the reduced power consumption and elimination of safety problems. However, the temperature stability should be taken into account, especially for the RT sensors. Maybe a little higher temperature (<100 °C) would be better to render the required stability.

Humidity of the environment has also been taken into account by researchers as an important factor to the hydrogen sensing of SMOs. As reported by Zhang and Gong, *et al.*, water absorbing on the surface of SMOs will lower the sensitivity of gas sensors [61,62]. Firstly, the reaction between the surface oxygen and the water molecules leads to a decrease in baseline resistance of the gas sensor, and results in a decrease of the sensitivity. Secondly, water molecules adsorbed on the surface lead to lower surface absorption site for chemisorption of oxygen species, which results in lower sensitivity. Besides, high humidity may also result in poor repeatability of the sensing performance. Therefore, the influence of humidity should be restrained or eliminated either by the design of material and device structure or by inducing the humidity compensation to the sensitivity.

The long-term stability is a key factor for gas sensors, but it is always been ignored by researchers, which has limited the practical applications of the sensors, especially when the sensors are operating at elevated temperatures. The grain size, necks and boundaries as well as the surface defects would be changed during the long-term operation at high temperatures, which leads to the variation of hydrogen response under the same condition and decreases the sensor stability [16]. Therefore, nanostructures with single crystalline structure such as single crystalline NWs possess better long-term stabilities than polycrystalline nanostructures, especially when the sensor is based on individual single crystalline NWs with no nanojunctions. The better long-term stability of NW-based gas sensors has already been found for other kinds of gas sensors, such as the randomly oriented single crystal SnO_2_ NWs invested by Sysoev and co-workers [63], and the individual NW-based sensors demonstrated by Hernadez-Ramirez *et al.* [64].

## 3. Hydrogen Sensors Based on SMO Thin Films and 1D Nanostructures

### 3.1. Thin Films

Nanocrystalline thin films, which are two-dimensional nanostructures, have already been investigated as gas sensing materials for many years. Recently, SMO thin films have attracted considerable interest as hydrogen sensing materials due to their higher specific surface area and smaller grain size than bulk materials, which can lead to higher response, lower operating temperatures and fast response processes. Moreover, along with the development of synthesis techniques, hydrogen sensors based on SMOs thin films exhibit good compatibility with integrated circuits for building integrated sensors. In recent years, a lot of factors have been investigated for improving the sensing performance of thin film hydrogen sensors, e.g., particle size, porosity, orientation, doping effect, noble metal compositing and electrode architecture, *etc.* In this section, the review of studies on hydrogen sensing of SMO thin film are mainly focused on the reports during the last five years. Among numerous SMOs, SnO_2_, TiO_2_ and ZnO thin films were mostly investigated, while In_2_O_3_, WO_3_, CuO, and NiO thin films were also employed as hydrogen sensing materials.

**Figure 6.**
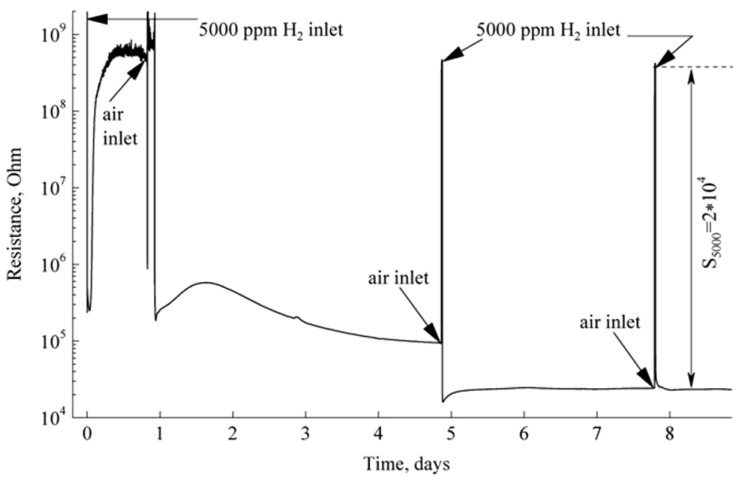
The resistance changes of sol-gel derived SnO_2_ thin film sensor during long-term exposure in hydrogen containing environment at 150 °C (reprinted from [66] with permission from Elsevier)

Most of the SMO thin film hydrogen sensors are resistance type ones, which operate based on the variation of conductivity of the SMOs. Table 1 lists the recent studies on the hydrogen sensing performance of the resistance based SMOs. We have made a suitable transformation to these data, especially the sensitivity which were uniformed to be *S* = *R*_0_/*R*_g_. Thus, the sensor responses from other literature in Table 1 are comparable. For instance, Adamyan and co-workers have reported the hydrogen sensing performance of the nanocrystalline SnO_2_ thin films with high sensitivity derived from a sol-gel annealing process, which is a common method for fabricating nanocrystalline oxide thin films and ensures the high-temperature stability of the nanocrystalline grains [65,66]. They suggested that pulse heating treatment during the sensing process could reduce the influence of environmental humidity, improve the stability and reduce the performance drift. As shown in Figure 6, they also studied the sensor parameters in real time during short- and long-term operation, which is always ignored by researchers. The sensing parameters were improved by a long-term treatment with an increase of sensitivity on more than two orders of magnitude. They attributed the improvement of performance to the diminished oxygen adsorption on defects during subsequent sensor operation in a hydrogen atmosphere. The diminishing oxygen adsorption on defects can reduce the local fields of defects and increase the oxygen adsorption on regular lattice atoms of the surface, which can enhance the hydrogen response. This work has had a great impact on emphasizing the importance of long-term stability of SMOs nanostructure-based hydrogen sensors.

The sensing performance can be modified by the intrinsic character of the SMOs, e.g., the specific surface area. A feasible method to increase the specific surface area of the SMOs thin films is to employ nanoporous thin films as sensing materials, for instance, the TiO_2_ nanoporous thin films and nanotube arrays. Chen and co-workers have reported a nanoporous TiO_2_ rutile thin film made by using a nanoporous anodized aluminum oxide (AAO)-assisted fabrication process. They found that the sensitivity was significantly enhanced by the increased specific surface area of the TiO_2_ thin film due to the shaping of the porous AAO substrate [11]. Similar results of TiO_2_ and Nb_2_O_5_ nanoporous materials were also reported by Sadek [67] and Hyodo [14] *et al.*

**Figure 7.**
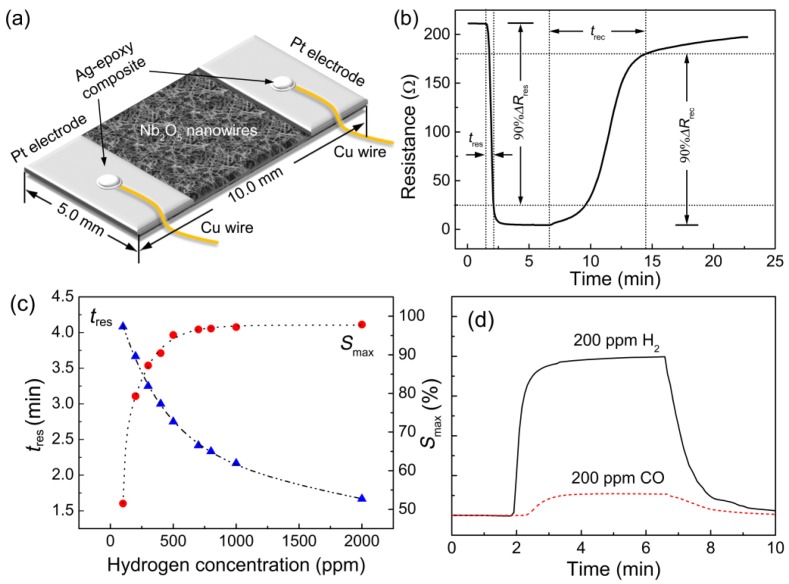
The RT hydrogen sensing properties of Nb_2_O_5_ NW thin films. (**a**) Schematic diagram; (**b**) response to 2,000 ppm of hydrogen in air; (**c**) hydrogen concentration dependent response; (**d**) selective response against CO (reprinted from [69] with permission from Elsevier).

Besides the porous thin films, thin films composed of NWs with high specific surface area can also be used for hydrogen gas sensing. Hung and co-workers have investigated the hydrogen gas sensing properties of ZnO wire-like thin films synthesized by thermal oxidation [68]. The films exhibited a sensitive and fast hydrogen response to 200 ppm of hydrogen gas in air at 200 °C. Recently, our group has demonstrated a RT hydrogen sensor based on Nb_2_O_5_ NW thin films [69]. The NW thin film was synthesized by a thermal oxidation of Nb foil at 900 °C, which was composed of interlacing Nb_2_O_5_ NWs with 30–50 nm in diameter. As shown in Figure 7, the sensor shows a sensitive response to 100–2,000 ppm of hydrogen gas in air at RT. The response time (<5 min), however, is still in need of further improvement, which may be due to the slower response from the grain boundaries at the contact site between the NWs than that from surface reaction under RT.

Moreover, the hydrogen sensing behavior can also be affected by the orientation of the SMO thin films. For instance, Choi *et al.* have reported the hydrogen sensing properties of highly oriented SnO_2_ thin films. They found that the (101) oriented SnO_2_ films grown on (112̄0) Al_2_O_3_ substrates exhibited higher hydrogen gas response (*S* = *R*_0_/*R*_g_) of ∼300 to 10,000 ppm of hydrogen than (002) and (101) oriented ones grown on (101̄0) and (11̄02) Al_2_O_3_ substrates, respectively. According to the Auger electron spectroscopy (AES) and X-ray photoelectron spectroscopy (XPS) analysis results, the large difference on the sensitivity between two (101) oriented films grown on (112̄0) and (11̄02) Al_2_O_3_ should be due to the presence of the Sn^2+^ component (SnO) at the near surface region along with the Sn^4+^ component (SnO_2_) by the Auger electron spectroscopy [70].

Besides, the grain boundaries of TiO_2_ thin film hydrogen sensors were also taken into account by Ling and co-workers. They fabricated TiO_2_ thin films by a micro-arc oxidation process and found that the maximum gas response of the sensor is estimated to be 2.5 and the response time is 45 s at 250 °C, but no gas response was seen above 300 °C. According to the electrochemical impedance spectroscopy (EIS) analysis results, the authors suggested that the lower gas response at higher temperature was caused by the disappearance of the depletion layers at the grain boundaries [71].

**Figure 8.**
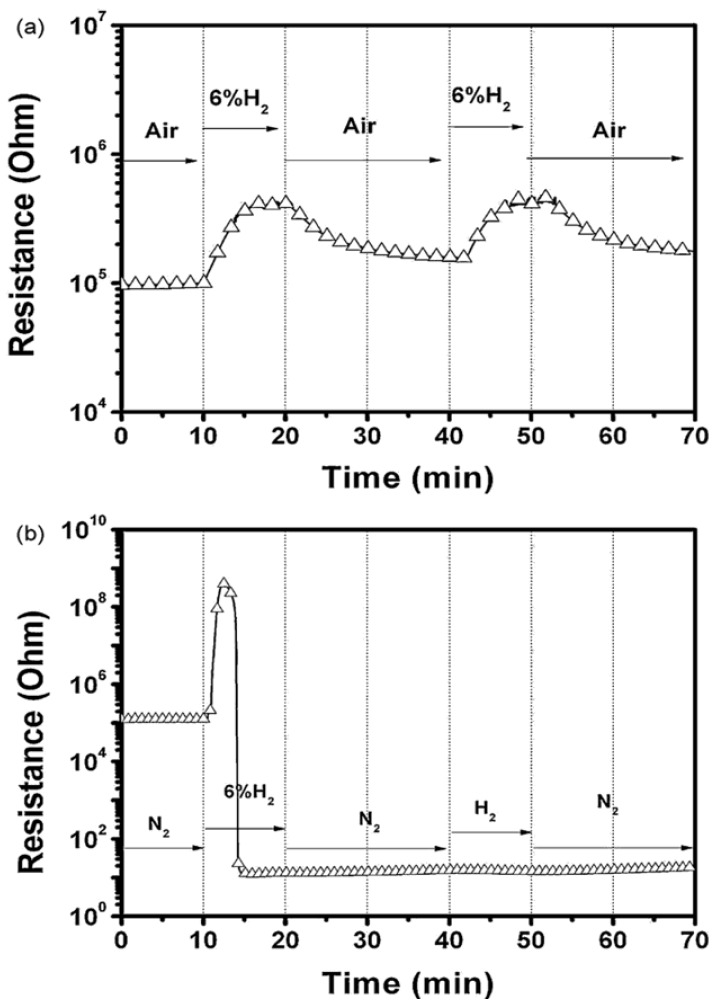
The response-and-recovery behaviors of CuO thin film oxidized at 400 °C in different dilution gases of (**a**) dry air and (**b**) nitrogen at 250 °C (reprinted from [72] with permission from Elsevier).

Most SMOs used as hydrogen sensing materials are n-type semiconductors, while CuO and NiO with *p*-type conduction can also be employed for hydrogen sensing. Hoa *et al.* synthesized p-type semiconducting CuO thin films by deposition and thermal oxidation of Cu on SiO_2_ substrates [72]. As reported, the p-type semiconducting property is due to the Cu vacancies in the crystal structure (Cu_1−δ_O) [73]. The CuO thin films showed increased resistance upon exposure to a hydrogen-containing environment. Except for the first intense hydrogen response, however, the sensor showed no response at all after the hydrogen dilution by N_2_ gas, as shown in Figure 8. This result demonstrated the key role of oxygen adsorbed on the surfaces of the CuO sensor materials, and provided a typical example showing no reaction with hydrogen without mediation via oxygen. Steinebach and co-workers also reported the p-type hydrogen sensing behavior of NiO thin films at elevated temperatures (T > 500 °C) [74]. They found that the gas response of the NiO thin films was correlated to the surface roughness. A highest response could be obtained at the thin film with highest surface roughness due to the high specific surface area offering more surface adsorption site for oxygen from the gas phase. In addition, a tunable gas sensing behavior of ZnO thin films between p-type and n-type were reported by Kobrinsky and co-workers [75]. This work indicates that the exposure to hydrogen gas may invert the sensor between p- and n-type in a fast and reversible manner.

**Figure 9.**
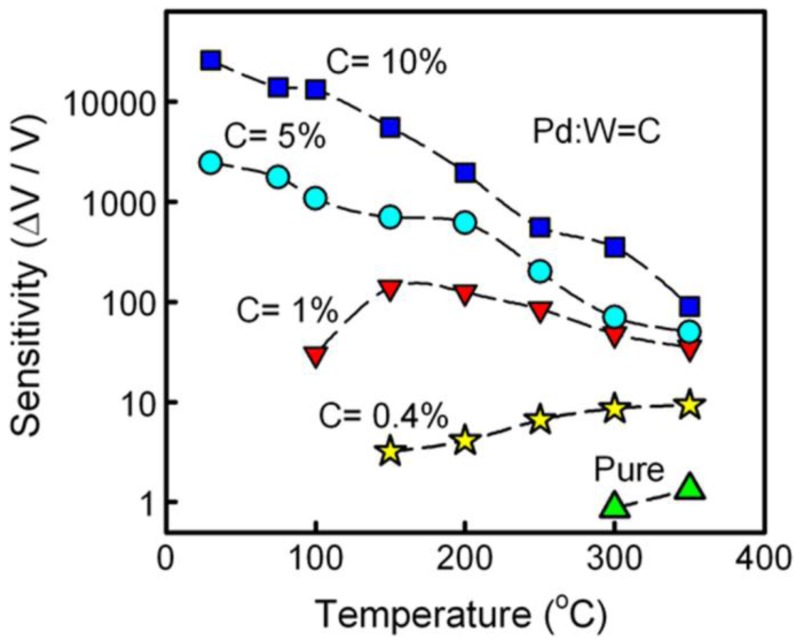
The temperature dependent sensitivity to 1,300 ppm of hydrogen gas in air of Pd/WO_3_ thin film (reprinted from [78] with permission from Elsevier).

Besides the intrinsic character of SMO thin films, the hydrogen sensing properties can also be improved by other approaches, e.g., by doping, compositing with noble metal nanoparticles, as well as adjusting of electrode parameters, *etc.* Among them, doping is an effective method to modify the sensing performance of SMOs. Galstyan *et al.* investigated the hydrogen sensor of Al-doped ZnO thin film, which have high sensitivity to low concentrations of hydrogen in air (1,000 ppm) [76]. Liu *et al.* have reported a great enhancement of the hydrogen sensing performance of ZnO thin films by Mg doping, and suggested that the higher sensitivity of Mg-doped ZnO thin films should be due to the larger resistance [77]. Moreover, Palladium (Pd) has already been used for doping in SMOs due to its outstanding catalytic properties. Shen *et al.* have reported Pd-doped SnO_2_ thin films with columnar nanostructures and their hydrogen sensing properties [60]. The thin films were deposited by reactive magnetron sputtering of a Pd/Sn target. Their results proved that Pd-doping can enhance the sensitivity of the SnO_2_ thin film to hydrogen gas at 300 °C, which was attributed to the so-called chemical and electronic effects. Moreover, the columnar nanostructures were also considered to play an important role for improving the sensing performance due to the higher specific surface area and increasing of the surface Schottky-barrier-limited electron transportation. Fardindoost *et al.* also demonstrated the hydrogen sensing of Pd-doped WO_3_ thin films prepared by a sol-gel process [78]. They suggested that Pd can modify the growth kinetic of WO_3_ nanoparticles by reducing the crystallite size and therefore improve the hydrogen sensitivity. As shown in Figure 9, the hydrogen sensitivity increased as increasing the Pd doping concentration in the whole temperature range (30 to 350 °C), which could be attributed to a decrease in WO_3_ crystal size and the effect of Pd catalyst in performing electronic sensitization mechanism. In addition, the sensitivity increases by decreasing the operating temperature. They attributed that to a reduction of the activation energy between the WO_3_ surface and the hydrogen gas in the presence of Pd (PdO). The sensitivity is up to 2.5 × 10^4^ at RT under exposure to 1,000 ppm of hydrogen in air, and the sensor exhibited its optimum sensing performance at 100 °C due to the better response and recovery time as well as the better temperature stability to the environment. Similar results were also reported by Zhang *et al.* of their Pt-doped WO_3_ thin films.

Besides doping effects, noble metal nanoparticles (Pd, Pt and Au, *etc.*) were also used for improving the gas sensing performance of SMOs due to their catalytic properties. Fasaki *et al.* have demonstrated the effect of Au and Pt nanoclusters on the hydrogen sensing properties of SnO_2_ thin films [79]. The addition of metal nanoparticles was found to decrease the detection limit and the operating temperature (from 180 °C to 85 °C), while increasing the sensing response signal. In the presence of Au or Pt nanoclusters on the surface, the hydrogen gas would split up into hydrogen atoms by the catalytic process. The rapid transfer of the adsorbed dissociated hydrogen atoms mediated by the metal nanoclusters, which is known as a spill-over effect, leads to an increase in the rate of interaction with SnO_2_, and results in the enhanced hydrogen sensing performance.

Recently, carbon nanotubes were used as doping additives in SMO thin films. Gong and co-workers fabricated micromachined sol-gel single wall carbon nanotube (SWCNT)/SnO_2_ nanocomposite thin films and found that the hydrogen sensing performance of the SWCNT/SnO_2_ sensors was better than that of the pure SnO_2_ sensor, including higher sensitivity, lower working temperature, and shorter response/recovery time [80]. The greatly improved performances are mainly attributed to the effective gas accessing nanopasses through SWCNT plus the smaller distance between adjacent gas accessing boundaries formed by the distribution of tiny SWCNTs. Wongchoosuk and co-workers reported their research works about the MWCNT-doped WO_3_ thin films with higher sensitivity and selectivity to hydrogen, as well as reduced operating temperatures [81]. They suggested that the creation of nanochannels and formation of *p-n* heterojunctions were responsible for the enhanced sensing behavior.

As reported by Seal and co-workers, the electrode architecture is important to the hydrogen sensing performance of the SMO thin film sensors, because of the increased activation energy that may impede the overall sensor response at lower temperatures (especially RT) [82–84]. Both the experimental results and the theoretical study using a diffusion reaction model suggested the electrode spacing-dependent (2–20 μm) response kinetics of the nano-micro-integrated hydrogen sensor at RT, which should be due to the reduced path length for free electrons

Although most investigations have focused on improvement of sensing performance of the resistance type sensors in recent years, there were also some studies on the hydrogen sensing of Schottky diode, MOS capacitor, optical and acoustic type SMO thin film sensors. For instance, Shafiei, Yu and co-workers have demonstrated the Pt/nanostructure ZnO and Pt/ZnO/SiC based Schottky diode based hydrogen sensors [33,85,86]. They all found that a larger voltage shift was produced by the sensor under reverse bias conditions than under forward bias conditions. As explained by Shafiei, this is caused by the increase in free carrier concentration and the decrease in permittivity, which can be enhanced by the augmented localized electric fields produced by the nanostructures. TiO_2_ and Nb_2_O_5_ nanoporous thin film were also employed as semiconductor layers to fabricate Schottky diode type hydrogen sensors, which exhibited outstanding sensing properties due to the high specific surface area and catalytic behavior of Pd electrodes [9,14]. Yadav *et al.* reported a Pd/TiO_2_/Si MOS capacitor hydrogen sensor with very large parallel shift in *C-V* and *G-V* characteristics, which appears to be basically due to the high polarizability of TiO_2_ lattice and increase in interface trap charges in presence of hydrogen gas and could be used for RT hydrogen sensing [46].

Acoustic wave hydrogen sensors have advantages that allow for remote wireless operation and possess high potential application possibility on passive sensors. Recently, Phan and Chung reported their research work on a SAW hydrogen sensor based on ZnO nanoparticle thin films [87]. They deposited a Pt/ZnO sensing layer on the SAW delay line sensor based interdigital electrodes/AlN/Si structure. A maximum frequency down shift of 55 kHz was observed when the layered sensor was exposed to 10,000 ppm of hydrogen gas at RT, which is three times higher than the conventional SAW sensor.

Unlike the electrical hydrogen sensors, the optical hydrogen sensors are fabricated by the coating of SMO thin films on the surface of the optical fiber. Yan and co-workers have demonstrated a SnO_2_ thin film-based optical fiber hydrogen sensor [88]. The SnO_2_ coating induced an optical absorption peak centering at around 320 nm when the SnO_2_-coated optical fiber were exposed to H_2_-N_2_ gas at a temperature higher than 300 °C. The absorbance increased as the hydrogen concentration increased. Besides, high hydrogen selectivity was also observed with no optical absorption response to other reducing gases including CO and CH_4_. Moreover, TiO_2_ thin films with the assistance of Pd were also used as SMOs in optical hydrogen sensors. Yang and co-workers demonstrated an optical fiber hydrogen sensor based on side-polished fibers with Pd/WO_3_ thin film coating [89]. This sensor operated based on the evanescent field interaction mechanism, and showed an increase of transmitted power when hydrogen concentration increases. In addition, sensors based on multimode fibers shows higher sensitivity but lower response linearity to hydrogen concentration than sensors based on single mode fibers.

### 3.2. 1D Nanostructures

Compared to SMO thin films, 1D SMO nanostructures such as NWs, NRs, nanobelts and nanotubes, possess higher specific surface areas and unique 1D structures [90,91], which lead to better gas sensing properties including ultra-high sensitivity, fast response, low working temperature and LOD, as well as good long-term operation performances [4,15,16]. In recent years, the number of investigations on hydrogen sensors based on 1D SMO nanostructures has grown rapidly with the development of large scale synthesis processes and micro-/nano-scale fabrication techniques. Several kinds of sensor architectures were also developed based on the difference of fabrication techniques to investigate the hydrogen gas sensing behavior of individual or aligned multiple 1D nanostructures.

#### 3.2.1. Individual SMO 1D Nanostructures

By means of the rapid development of nano-fabrication technique, hydrogen sensors based on individual NWs were fabricated by letting the NW bridging crossover the nano-scale electrodes either through the focused ion beam (FIB), electron beam lithography (EBL) process or by the dielectrophoresis (DEP) controlling. As a result, nearly all these hydrogen sensors are based on the resistance variation with the exposure to hydrogen containing atmosphere. Table 2 lists the sensing performance of recently developed hydrogen sensors based on individual SMO 1D nanostructures.

A similar feature of the resistance-type hydrogen sensors based on individual SMO 1D nanostructure is the outstanding RT operation performance. For instance, Fields and co-workers have reported their RT hydrogen sensor with low power consumption, which is based on the individual SnO_2_ NW connected by a pair of RuO_2_ nano-electrodes [92]. Without any functionalization with catalyst, the sensor shows a response of *S* = 60% (*S* = Δ*R/R*_0_) at 25 °C to 20,000 ppm of hydrogen in air with a response and recovery time of approximately 220 s. The RT sensing performance of the unfunctionalized SnO_2_ NW is much better than that of the pure SnO_2_ nanostructured thin films, and is believed to be further improved by surface functionalization of the catalyst.

Lupan and co-workers have reported a series of research works about hydrogen sensors based on individual ZnO 1D nanostructurea connected by nano-electrodes fabricated through a FIB process [93–96]. The hydrogen gas nanosensor based on two-terminated single ZnO NR (tripod/dipod) that they reported in 2007 and 2008 exhibited a response of 2% and 4% (*S* = Δ*R/R*_0_) with a short response time of 1 min to 150 and 200 ppm of hydrogen in air under RT, respectively [93,94]. Furthermore, the sensitivity to several common gases like O_2_, CH_4_, CO, and LPG were less than 0.02% and 0.25% under the same conditions respectively, which indicated the high selectivity of hydrogen. Owing to the RT operation, the working power is very low (<5 μW), compared with thin film based sensors.

**Figure 10.**
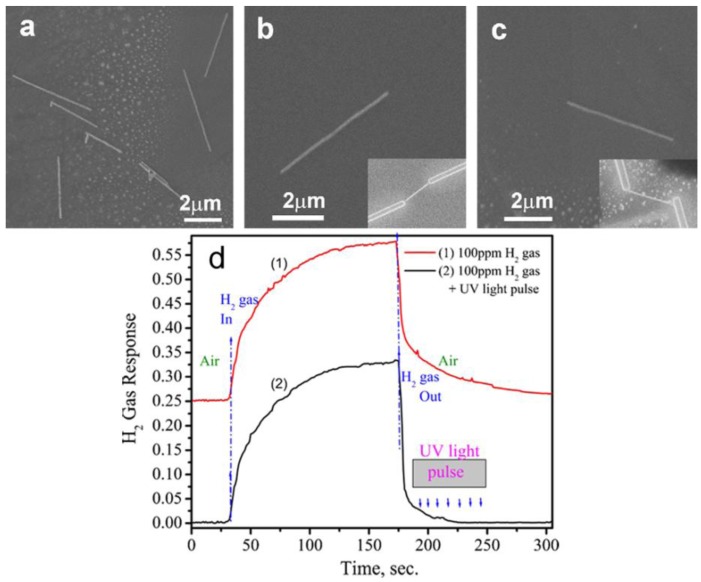
The SEM image of dispersed (**a**) and connect ZnO NWs with 200 nm (**b**) and 100 nm (**c**) in diameter; (**d**) Transient response of the 100 nm ZnO NW-based sensor to 100 ppm of H_2_ gas at RT (22 °C) (reprinted from reference [96] with permission from Elsevier).

They also fabricated hydrogen nanosensors based on individual ZnO NWs connected by Cr/Au nanoelectrodes on both ends [95,96]. They investigated the photoluminescence spectra of the thermally treated ZnO NWs in a hydrogen environment at 400 °C, and found that the concentration of defects responsible for visible emission from ZnO NW was reduced by annealing in a hydrogen atmosphere, which means a passivation of recombination centers. The author indicated that this effect may lead to the reduction of hydrogen response and should be taken into account when analyzing the long-term stability of the NW based sensors. Therefore, the development of RT sensors is necessary. Besides the outstanding RT performance on hydrogen sensing as shown in Figure 10, the ZnO NW sensor also exhibited enhanced recovery behavior by employing ultraviolet (UV) light illumination instead of heat treatment to facilitate the desorption of gas species. The outstanding hydrogen performance can be attributed to: (a) the small diameter of ZnO NWs that means high specific surface area and leads to more surface atoms for surface reaction; (b) the small diameter of ZnO NWs, comparable to the Debye length (a measure of the field penetration into the bulk) that causes the electronic properties to be more influenced by adsorption-desorption processes at the surface; (c) the increased electron and hole diffusion rate to the surface that allows the analyte to be rapidly photo-desorbed from the surface even at RT under UV light pulse; (d) the stoichiometric composition of ZnO NW grown by chemical vapor deposition (CVD) with a higher level of crystallinity than multigranular oxides. The author suggested that the operation mode of ZnO NW devices can be controlled by the modulation of surface states through surface morphology engineering and size control.

Recently, DEP controlling has been gradually introduced for fabricating individual NW and NW-network based devices. Owing to the small diameter and high aspect ratio, NWs are very easy to control for the alignment between micro- or nano-scale electrodes by adjusting the DEP parameters. Huang *et al.* have demonstrated a hydrogen sensor based on SnO_2_ single NR, which is aligned by DEP controlling and fixed by Pt stripes onto the microelectrodes [97]. This sensor shows a fast and selective response to 100 ppm of hydrogen at RT, but shows its best performance at 250 °C. The good sensing performance of the NR sensors can be attributed to the intrinsically small dimensions of the NR, comparable to the depletion layer. Although DEP can be used for fabricating individual NW/NR based sensors, the control accuracy is much lower than with the FIB or EBL processes, therefore, it is mostly used for fabricating NW/NR networks.

In addition to the FIB and DEP controlling methods, Rout and co-workers have demonstrated the hydrogen sensing characteristics of single NWs of ZnO, TiO_2_ and WO_2.72_ by contact mode atomic force microscopy (AFM), which actually represents the response from the radial direction of the NWs. All SMO NWs investigated in this work showed fast and sensitive response to 1,000 ppm of hydrogen gases [98].

**Figure 11.**
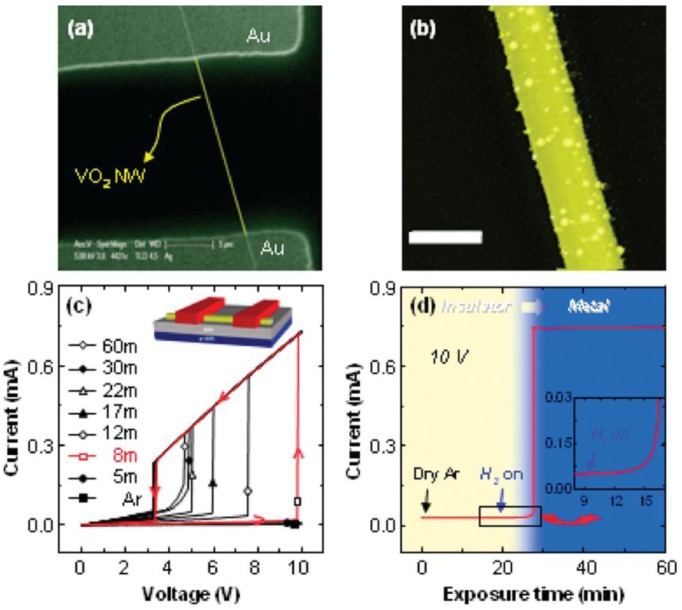
(**a**) SEM image of an individual VO_2_ NW device with appropriate Ohmic contacts (**b**) SEM image of a Pd-decorated VO_2_ NW; (**c**) *I-V* curves obtained at 50 °C for Pd-decorated VO_2_ NW after various exposure times to hydrogen gas (5 sccm), added to the background argon stream (10 sccm); (**d**) The change in current for a Pd-decorated VO_2_ NW biased at 10 V as a function of time of exposure to hydrogen gas (reprinted from [99] with permission from The American Chemical Society).

Baik and co-workers have reported a hydrogen sensor based on the metal-insulator transition (MIT) of Pd-sensitized VO_2_ NW, as shown in Figure 11 [99]. By biasing the NW judiciously so that its temperature is just below the metal-insulator transition temperature, even trace amounts of hydrogen induce the metal-insulator transition, producing an almost three order of magnitude increase in the current through the NW. The authors suggested that the large change in the MIT transition temperature of VO_2_ may result from the formation of hydrogen interstitials or from the formation of adsorbed OH, which act as electron donors that increase the carrier density in the conduction band. Two processes during hydrogen response were illustrated. During a rapid initial process, the insulator to metal transition temperature is decreased by >10 °C even when exposed to trace amounts of hydrogen gas. Subsequently, hydrogen continues to diffuse into the VO_2_ for several hours before saturation is achieved with only a modest change in the insulator to metal transition temperature but with a significant increase in the conductivity. The two time scales over which hydrogen-related processes occur in VO_2_ likely signal the involvement of two distinct mechanisms influencing the electronic structure of the material, one of which involves electron-phonon coupling pursuant to the modification of the vibrational normal modes of the solid by the introduction of H as an impurity.

#### 3.2.2. Multiple SMO 1D Nanostructures

Unlike the individual 1D nanostructure based sensors, sensors based on multiple SMO 1D nanostructures do not need precise nano-fabrication techniques to connect the nanostructures. The relatively simple fabrication technique may greatly decrease the manufacturing costs and increase the repeatability of the sensors with outstanding sensing performance, which is more suitable for practical applications. Table 3 lists the hydrogen sensing performance of recently developed 1D SMO nanostructure networks with resistance type response.

**Figure 12.**
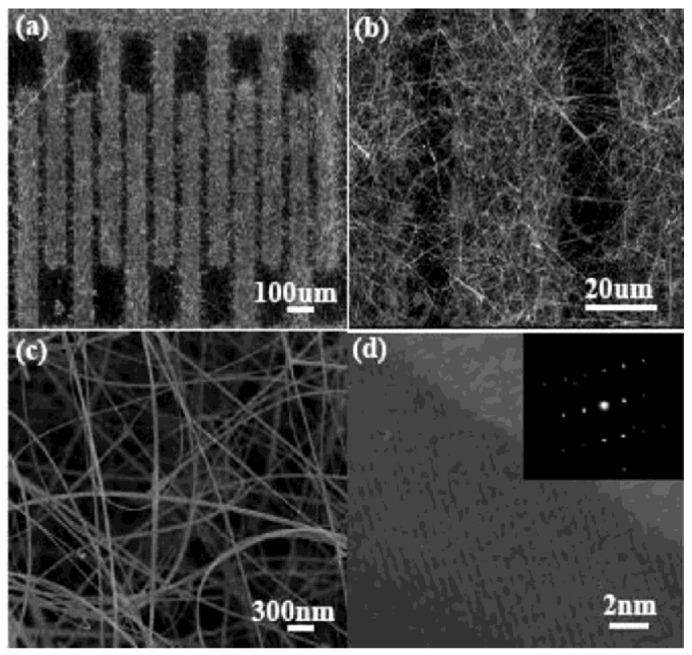
The SEM and TEM images of the as-fabricated SnO_2_ NW gas sensor. low magnifying (**a**) and high magnifying (**b**) SEM images; (**c**) SEM image of the interspace of electrodes; (**d**) the HRTEM image of one SnO_2_ NW and the corresponding SAD pattern (inset) (reprinted from [100] with permission from The American Chemical Society).

Wang and co-workers have demonstrated an on-chip self-assembled SnO_2_ NW hydrogen sensor with SnO_2_ NWs extending from one side to the other of the comb-shape interdigital electrodes, which is shown in Figure 12 [100]. With the decreasing gap between the electrodes, the spatial density of the SnO_2_ NWs is higher. The sensor response is enhanced because of the geometric effect associated with the high electrode spatial density. Thus, the sensor response of the SnO_2_ NW sensor with a gap of 20 μm is higher than that of the SnO_2_ NR sensor with a gap of 50 μm. In addition, the SnO_2_ NW sensor has better gas sensing than that of the SnO_2_ NR sensor due to the nano-size effect and could be more promising in practical applications. Several other gas sensors with similar structure were also reported [101–103].

Another commonly used fabrication method of SMO 1D nanostructure network hydrogen sensor is realized by transferring as-synthesized SMO 1D nanostructures by droplets onto a pair of interdigitated electrodes. Duc Hoa *et al.* synthesized p-type semiconducting CuO NWs by thermal oxidation of copper wire in air and transferred the NWs onto a pair of interdigitated electrodes to fabricate a CuO NW based hydrogen sensor [104]. The sensor could detect a wide range of gases, including 1% H_2_, 60 ppm CO, 60 ppm NH_3_, and 60 ppm NO_x_ at elevated temperatures (200–300 °C). Qurashi and co-workers have reported their hydrogen sensors based on In_2_O_3_ NWs/nanoneedles and ZnO NW/NR networks fabricated by this process [105–109]. Among them, In_2_O_3_ NWs and nanoneedles grown along the (110) direction were synthesized by a catalyst-supported CVD process, and showed fast, reliable and stable hydrogen responses at elevated temperatures (200 °C) [108]. The In_2_O_3_ NWs showed better sensing performance than nanoneedles, which was attributed to the smaller diameter and high surface-to-volume ratio of the NWs. The authors also suggested that the number of interconnections between the two electrode fingers should be taken into account.

The nanojunction effect to the hydrogen sensing behavior in the interconnected multiple ZnO NWs were investigated by Khan *et al.* [110]. They used the DEP process to make the ZnO NWs positioned and aligned between two Ti and Au electrodes and deposited a top contact electrode of Ti and Au for better contact. By controlling the DEP force, they obtained both single and multiple NW devices. Both devices showed n-type semiconductor characteristics, while the multiple NW devices showed greatly enhanced conductance due to increased channels. The sensor response was higher for the multiple ZnO NWs than the response for the single ZnO NW over the entire range of hydrogen concentration. The author attributed that to the nanojunctions acting as potential barriers for electron flow, which decreased as the NW was exposed to the reducing gas, resulting in an increase in the current flow. However, the nanojunction effect also affected the recovery time, which can be attributed to the significant diffusion resistance in the recovery period because of the low diffusivity of oxygen molecules. They suggested that the potential barrier modulation of multiple NWs was more efficient than the modulation of the surface depletion of the single ZnO NW in gas sensing.

**Figure 13.**
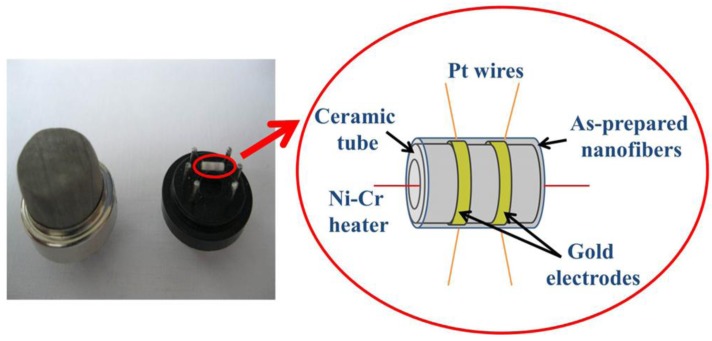
Schematic illustration of the ceramic-tube gas sensor with nanofibers coating on the surface (reprinted from [112] with permission from Elsevier).

As mentioned above, doping is an efficient method for improving the gas sensing performance of SMO thin films. Furthermore, it is also suitable for improving the sensitivity and response time for SMO 1D nanostructures. For instance, Shen *et al.* have compared the hydrogen sensing properties of undoped and Pt-doped SnO_2_ NWs, and found that Pt doping not only improves the sensitivity, but also lowers the operating temperature at which the sensitivity is maximized [111]. Moreover, the sensitivities of Pd-doped SnO_2_ NWs are higher than those of Pt-doped SnO_2_ NWs, especially in the temperature range of 50–250 °C. The difference in the sensing properties of two types of impurity-doped NWs may be attributed to two different effects of “chemical sensitization mechanism” from Pt-doping and “electronic sensitization mechanism” from Pd-doping, respectively. Moreover, doping is more commonly realized for SMO nanofibers synthesized by electrospinning and calcination procedures. The nanofibers were always spin-coated on a ceramic tube with a pair of Au electrodes printed on the surface and a Pt heating wire inserted into the tube to form a side-heated gas sensor, as shown in Figure 13 [112]. For example, Liu, Zhang and co-workers have demonstrated the enhanced hydrogen sensing performance of Pd and Co doped SnO_2_ nanofibers [113,114]. When Co was doped in SnO_2_, high response, short response and recovery times, and good selectivity were observed. The authors suggested that the Co_3_O_4_ grains in SnO_2_-rich materials will combine with SnO_2_ electronically by forming *p-n* junctions, which may explain the large sensor response. However, too much Co-doping would lead to decreased hydrogen response. Xu and co-workers have investigated the Al doping effect on the hydrogen sensing performance of electrospun SnO_2_ nanofibers [112]. Among them, the Al doping would generate more oxygen vacancies through the SnO_2_ crystals due to the partial substitution of Sn^4+^ cations with lower valence Al^3+^ cations together with their different ions radius. As increasing the doping concentration, external heterojunctions will be formed between Al_2_O_3_ and SnO_2_ owing to the expulsion of Al^3+^ ions from the SnO_2_ crystals. The Al_2_O_3_ nanoclusters could act as catalytic sites with spill over effect for redox processes and oxygen dissociation, which result in the enhanced sensing performance, however, the Al_2_O_3_ nanoclusters will reduce the surface area and amount of oxygen vacancies. The same authors also reported improved hydrogen monitoring properties based on p-NiO/n-SnO_2_ heterojunction composite nanofibers [115]. By adding NiO, *p-n* heterojunction is formed at the interface between NiO and SnO_2_. In this case, in an oxidizing atmosphere a thicker charge depletion layer is formed near the grain surface of SnO_2_ as a *p-n* junction. Therefore, a larger resistance change will obtained when the sensor was exposed to hydrogen containing atmosphere, which resulted in the higher sensitivity of the p-NiO/n-SnO_2_ heterojunction composite nanofibers than the pure SnO_2_ nanofibers.

Lee and co-workers have demonstrated an ultra-sensitive hydrogen sensor based on Pd-decorated SnO_2_ NWs, which can be operated under RT with the assistance of the “spill-over effect” induced by Pd nanoparticles.[17] The sensors showed ultra-high sensitivity (1.2 × 10^3^, *S* = Δ*R/R*_g_) and fast response time (<2 s) upon exposure to 10,000 ppm H_2_ at RT. These sensors were also found to enable a significant electrical conductance modulation upon exposure to extremely low concentrations (40 ppm) of H_2_ in the air.

**Figure 14.**
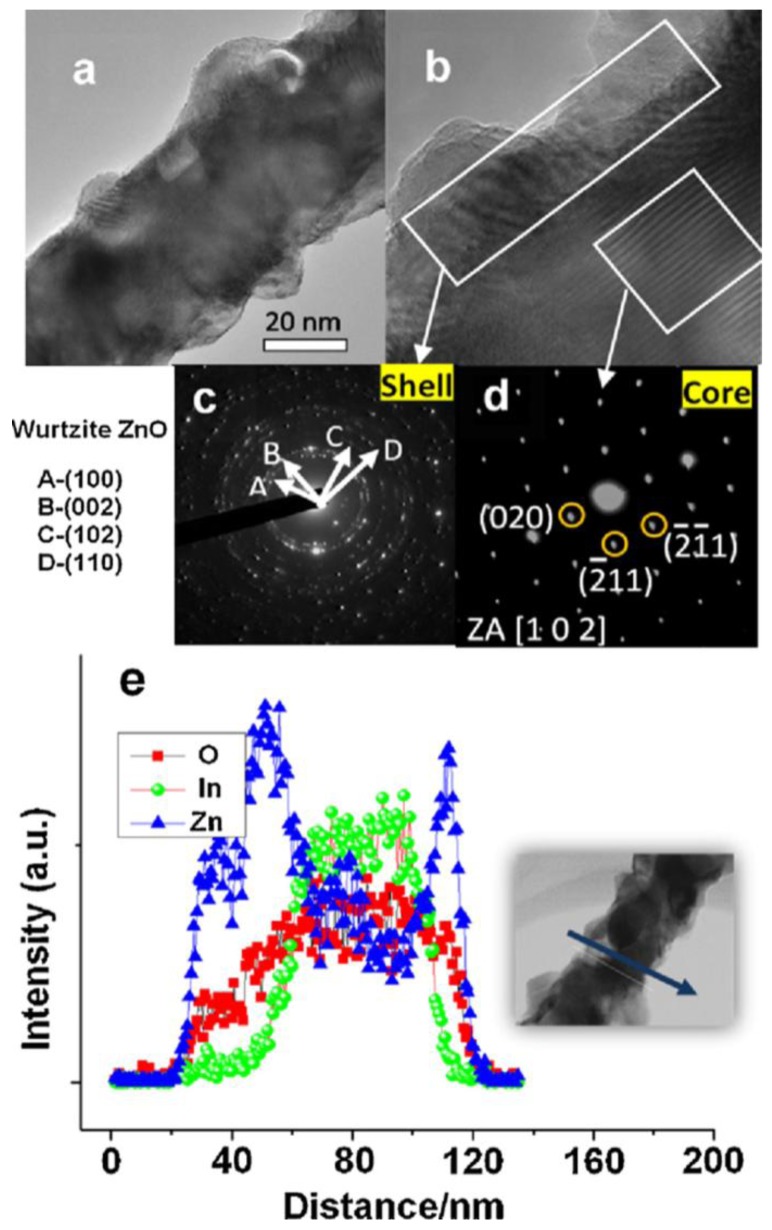
The TEM analysis of In_2_O_3_-ZnO core shell NW. (**a**) low magnifying TEM image; (**b**) HRTEM image; (**c**) SAED pattern obtained from the shell; (**d**) SAED pattern obtained from the core; (**e**) line profile obtained by TEM-EDS analysis (reprinted from [116] with permission from Elsevier).

Heterostructures of SMO 1D nanostructures have been used for improving hydrogen sensing properties due to their high surface area and hybrid properties. Singh *et al.* have synthesized In_2_O_3_-ZnO core-shell NWs by a two-step growth process, with polycrystalline ZnO coating on the single crystalline In_2_O_3_ NWs (shown in Figure 14) [116]. In_2_O_3_-ZnO core-shell NW networks showed higher response to reducing gases such as H_2_, CO, or ethanol compared to oxidizing gases such as NO_2_. Comparing with the In_2_O_3_ NW networks with only homointerfaces at the junctions, the In_2_O_3_-ZnO core-shell NW networks formed both homo- and hetero-junctions at the interfaces. The energy barrier induced by the homojunctions between In_2_O_3_ NWs has a profound effect on the gas sensing performance, which was also mentioned above. Moreover, the heterojunctions of In_2_O_3_ and ZnO lead to additional electron depletion layers due to the difference in work function. As the charge carriers need to travel through the interconnected NWs, the homojunction between two interconnected NWs, the grain boundaries in polycrystalline ZnO shell layer and the heterojucntion between In_2_O_3_ and ZnO must be passed through. When the sensor was exposed to hydrogen gases (or other reducing gases), all potential barriers were modulated, which may be the reason for explaining the higher sensing performance than In_2_O_3_ NW networks.

**Figure 15.**
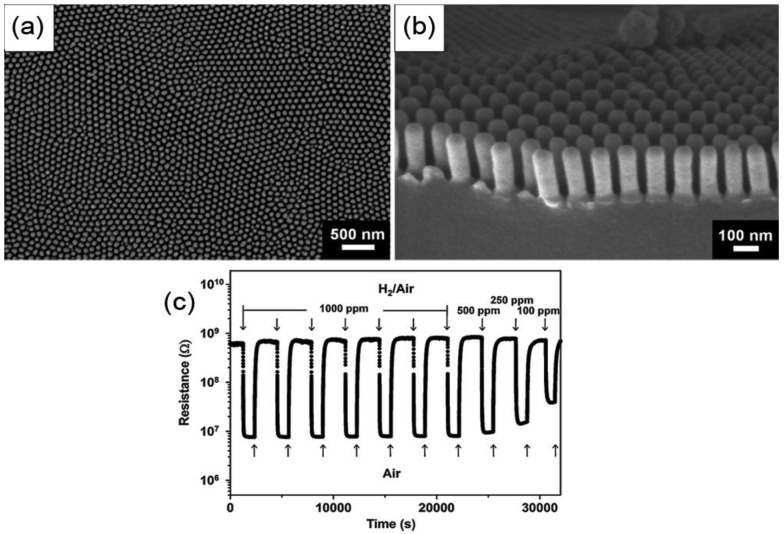
The SEM image (**a,b**) and hydrogen sensing performance (**c**) of vertically aligned anatase TiO_2_ nanotube arrays (reprinted from [12] with permission from Elsevier).

1D SMO nanostructure arrays, which are so-called quasi-1D nanostructures were also employed as hydrogen sensing materials due to their high specific surface area. Senik *et al.* have reported anodic oxidized highly-oriented TiO_2_ nanotubes for a hydrogen sensor, which exhibited high hydrogen sensitivity at RT but with a slow response time [10]. Jho and co-workers fabricated a hydrogen gas sensor by employing vertically aligned anatase TiO_2_ nanotube arrays prepared by AAO template-assisted atomic layer deposition method [12]. As shown in Figure 15, the sensor exhibited outstanding hydrogen sensing performance at low temperature (around 100 °C) in air environment with a maximum gas response (*S* = *R*_0_/*R*_g_) of 100.5 to 1,000 ppm hydrogen, as well as great stability, repeatability and selectivity against several reducing gases including NH_3_, CO, and C_2_H_5_OH. Moreover, the response time was extremely short (<1 s) even in the low temperature range. They attributed the outstanding performance to the vertically aligned TiO_2_ nanotubes locating apart from each other, which presumably resulted in the rapid gas diffusion in-between the nanotubes, and consequently allowed the hydrogen gas to quickly reach the entire surface of the nanotube. Besides, the hydrogen sensing properties of ZnO nanopillar and NR arrays have also been investigated [117,118].

There are some novel types of hydrogen sensors based on SMO 1D nanostructures which possess novel functions or device architectures compared with the NW-based sensors mentioned above. For instance, Liu *et al.* fabricated aligned ZnO nanotubes by electrospinning and sputtering techniques [119]. The sensor response (*S* = *R*_0_/*R*_g_) of the aligned nanotubes to 100 ppm H_2_ increases from 2.3 to 3.6 as the temperature increases from 200 to 400 °C. Beside, the sensor response of the ZnO nanotubes increases compared with that of the ZnO film prepared under the same condition. Zhu and co-workers have demonstrated the self-heated hydrogen sensor based on Pt-coated W_18_O_49_ NW networks with high sensitivity, good selectivity and low power consumption [120]. The hydrogen response obtained for the same concentration of hydrogen increases with increasing the applied voltage on the Pt-coated W_18_O_49_ NW network due to the increased self-heating of NWs, which lead to the increase of surface reactivity for redox reactions between adsorbed oxygen species and hydrogen atoms spilled over by Pt.

Recently, Tonezzer and Lacerda have reported a novel ZnO NW architecture integrated on a carbon microfiber textile [121]. Fast and sensitive hydrogen response was observed with an optimum response of 11 and response/recovery time of less than 12 s at 280 °C. Furthermore, the LOD is down to 4 ppm of hydrogen gas. Similar ZnO NRs fabrics were also reported by Lim and co-workers [122]. The fabric's response (*S* = Δ*R/R*_0_) after 10 min exposure of 1,000 ppm H_2_ gas was ∼83% after Pt-decoration compared to 27% without Pt catalyst. Both of the two novel structures give rise to higher surface area and flexibility of the device, but limit the application under high-temperature conditions. Moreover, the fact that lithographic techniques are not needed allows for large-scale and low-cost fabrication of gas sensors, as well as their application in the smart textile field.

## 4. Summary

In this article, we provide a comprehensive review of the hydrogen sensing properties of SMO nanostructures, including thin films and 1D nanostructures. According to their sensing behavior, the SMO sensors can be divided into resistance, Schottky diode, MOSFET, MOS capacitor, optical and acoustic types. Some critical issues such as sensitivity, response time, recovery time, gas selectivity, limit of detection (LOD), temperature/humidity influence and long-term stability were discussed. Nanostructured SMO thin film hydrogen sensors have the advantages of simple fabrication processes, good compatibility with integrated circuits for building integrated sensors, high sensitivity and short response/recovery times, *etc.* However, most thin film sensors still need to work at elevated temperatures, which results in poor long-term stability and high power consumption. The influences of grain size, porosity, orientation, doping and surface decoration as well as the device architecture on the sensing performance of hydrogen sensors have been widely investigated for improving gas selectivity and hydrogen response at low temperatures. Hydrogen sensors based on individual SMO 1D nanostructures can be obtained by employing nano-scale fabrication by FIB or EBL techniques, and the products exhibited ultra-sensitive, fast and highly selective responses to low concentration of hydrogen gas at RT, as well as outstanding long-term stability. Multiple SMO 1D nanostructures such as NW network-based hydrogen sensors fabricated by scalable micro-fabrication techniques were more suitable for practical applications than individual NWs. Interdigitated electrodes were always used for fabricating sensors based on single crystalline NWs, while ceramic tubes with a pair of Au electrodes were always used for fabricating sensors based on polycrystalline nanofibers. Doping, and noble metal decoration were also used for improving the sensing performance, while the effect of nanojunctions, heterostructures such as core-shell NWs were also investigated. Although a lot of efforts have been done for promoting the practical application of SMO nanostructure-based hydrogen sensors, there are some critical issues which still need to be resolved, e.g., the standard definition of sensor parameters and testing method, the investigation of standard LOD, long-term stabilities, as well as the validity, repeatability and calibration of the sensors.

## Figures and Tables

**Table 1.** The recent developed SMO thin film based resistance hydrogen sensors.

**Materials**	**Synthesis Method**	**Working Temp. (°C)**	**Detected Conc. (ppm)**	**Hydrogen response**				**Ref.**
				***S*_max_**	**Conc. (ppm)**	**t_response_**	**Temp. (°C)**	
SnO_2_	Sol-gel annealing	100–300	50–5,000	10^4^	5,000	<10 s	100	[65]
In_2_O_3_ doped SnO_2_	Sol-gel annealing	22	100–15,000	10^5^	15,000	tens of min.	22	[83]
(101)-SnO_2_	RF magnetron sputtering	550	300–10,000	300	10,000	<16 s	550	[70]
SWCNT doped SnO_2_	Sol-gel annealing	150–300	300–1,500	3	1,500	<5 s	250	[80]
Au or Pt enhanced SnO_2_	Sol-gel annealing	85–180	500–10,000	2	10,000	several min.	150	[79]
SnO_2_	Spray pyrolysis	250–400	1,000	3,040	1,000	2 s	350	[121]
Pd doped SnO_2_	Reactive Magnetron sputtering	50–300	10–1,000	85	1,000	several mins	200	[60]
SnO_2_	Sol-gel annealing	90–220	1,000	2,000	1,000	15 s	150	[66]
Al-doped ZnO	HF magnetron sputtering	40–100	1,000–5,000	10	1,000	10 min	100	[76]
ZnO wirelike thin film	Thermal oxidation	200	200	2.83	200	1.5 min	200	[68]
ZnO	Thermal oxidation	400	40–160	4,000	160	1,000 s	400	[122]
Mg-doped ZnO	PLD	150–300	5–5,000	50	5,000	5 min	300	[77]
Nanoporous TiO_2_	Thermal oxidation	500	5–500	10	500	10 s	500	[11]
Nanoporous TiO_2_	Anodic oxidation	100–300	1,200–10,000	1.24	10,000	-	225	[67]
Anatase TiO_2_	Micro-arc oxidation	100–300	1000	2.5	1,000	45 s	250	[71]
Nb_2_O_5_ NW thin film	Thermal oxidation	20	100–2,000	50	2,000	<2 min	20	[69]
MWCNT-doped WO3	Electron beam evaporation	200–400	100–50,000	3	1,000	-	350	[81]
Pd-doped WO_3_	Sol-gel annealing	20–350	1,000–1,300	10^4^	1,000	<100 s	20	[78]
Pt-doped WO_3_	RF magnetron sputtering	95–220	30–200	9.5	200	0.7 min	200	[123]
CuO	Thermal oxidation	300–800	60,000	3.72	60,000	5 min	250	[72]
NiO	PLD	25–250	30,000	1.16 (n) 1.76 (p)	30,000	10 min	125	[124]
NiO	Magnetron sputtering	300–650	500–10,000	190 (p)	5,000	5 min	400	[74]

**Table 2.** The hydrogen sensing performance of recent developed individual 1D SMO nanostructures with resistance type response.

**Materials**	**Working Temp. (°C)**	***S*_max_**	**Conc. (ppm)**	**t_response_**	**Ref.**
SnO_2_ nanobelt	RT-80	2 (at RT)	20,000	220 s	[92]
ZnO NR	RT	1.02	150	1 min	[93,94]
ZnO NW	RT	1.5	100	64 s	[95,96]
SnO_2_ NR	RT-350	13 (at 250 °C)	100	-	[97]
ZnO NR	RT	10	1,000	-	
TiO_2_ NR	RT	8	1,000	-	[98]
WO_2.72_ NR	RT	22	1,000	38s	

**Table 3:** The hydrogen sensing performance of recent developed 1D SMO nanostructure networks with resistance type response.

**Materials**	**Working Temp. (°C)**	***S*_max_ (Temp.)**	**Conc. (ppm)**	**t_response_**	**Ref.**
SnO_2_ NWs	200–300	3.3 (100 °C)	1,000	100 s	[100]
In_2_O_3_ nanopushpins	150–400	1.1 (250 °C)	500	35 s	[105]
In_2_O_3_ NRs	250	1.2	1,000	48 s	[108]
ZnO NWs	200	5.0	500	65 s	[106]
In_2_O_3_ NWs	150–400	1.1 (200 °C)	500	31 s	[109]
ZnO NWs	200	5.3	100	∼1 min	[110]
Pt coated W_18_O_49_ NWs	200	2.1	1,000	∼1 min	[120]
In_2_O_3_-ZnO core-shell NWs	100–400	7.0 (300 °C)	2,000	-	[116]
ZnO NRs textile	RT	5.9	1,000	10 min	[122]
ZnO NRs	325–350	2.0 (350 °C)	50	6 s	[127]
aligned ZnO nanotubes	200–300	3.6 (300 °C)	100	-	[119]
CuO NWs	200–300	2.0 (250 °C)	60,000	-	[104]
ZnO NWs on carbon microfiber	RT-320	5 (280 °C)	200	8 s	[121]
Pt-doped SnO_2_ NWs	RT-300	119 (100 °C)	1,000	-	[111]
Pd-decorated SnO_2_ NWs	RT	10^3^	10,000	2 s	[17]
